# 

**Published:** 1840-07

**Authors:** 


### CONTENTS OF No. XIX.

OF THE

### British and Foreign Medical Review.

JULY, 1840.

### Part First.—Analytical and Critical Reviews.

PAGE

Art. I.—1. Grundriss der gesammten Augenheilkunde. Von Dr. August Andreæ . . . . . . . 1

Elements of Ophthalmic Medicine and Surgery. By Dr. Augustus Andreæ.

2. Dissertatio Inauguralis medico-literaria complectens Conspectum Historicum Scholæ Clinicæ Ophthalmiatricæ Viennensis, &c. Auctore Anthonio Hardwiger . . . . . . . ib.

An Historical View of the Ophthalmological Clinic of Vienna. By Anthony Hardwiger.

3. On Single and Correct Vision, by means of Double and Inverted Images on the Retinæ. By W. P. Alison, m.d., f.r.s.e. . . . ib.

4. Contributions to the Physiology of Vision. By Charles Wheatstone, f.r.s. ib.

5. Klinische Darstellungen der Krankheiten und Bildungsfehler des menschlichen Auges, der Augenlider und der Thränenwerkzeuge nach eigenen Beobachtungen und Untersuchungen. Herausgegeben von Dr. Friedrich August von Ammon . . . . . . . ib.

Clinical Illustrations of the Diseases and Malformations of the Human Eye, the Eyelids, and Lachrymal Organs, founded on the Author's own Observations and Researches. By Dr. Frederick Augustus von Ammon.

6. De Iritide. Commentatio ab illust. Societate medico-practica quæ Lutetiæ Parisiorum floret, &c., premio aureo publice ornata. Scripsit Frid. Aug. ab Ammon, d.m. . . . . . . 2

On Iritis. An Essay to which a gold medal was awarded by the Society of Practical Medicine of Paris. By Dr. F. A. von Ammon.

7. Guide Pratique pour l'Etude et le Traitement des Maladies des Yeux. Par Ch. J. F. Carron du Villards . . . . . ib.

Practical Guide to the Study and Treatment of the Diseases of the Eyes. By Ch. J. F. Carron du Villards, m. & c.d.

8. Handbuch der Physiologie des Menschen. Von Dr. Johannes Müller, &c. ib.

Elements of Physiology. By J. Müller, m.d.

9. Cours d'Ophthalmologie, ou Traité complet des Maladies de l'Œil, professé publiquement à l'Ecole pratique de Médecine de Paris. Par M. Rognetta. ib.

Lectures on Ophthalmology, delivered publicly at the Practical School of Medicine of Paris; or a Complete Treatise on the Diseases of the Eye. By M. Rognetta, m. & c.d.

10. Handbuch der Augenheilkunde zum Gebrauche bei seinen Vorlesungen. Von Maximilian Joseph Chelius . . . . . ib.

Manual of Ophthalmic Medicine and Surgery, for the use of his Pupils. By Maximilian Joseph Chelius, m. & c.d.

11. Die sogenannte contagiöse oder ägyptische Augenentzündung. Eine Monographie. Von Burkard Eble, d.m. . . . . ib.

The so-called Contagious or Egyptian Ophthalmia. A Monography. By Burkard Eble, m.d.

12. An Introductory Lecture on the Anatomy, Physiology, and Diseases of the Eye. By Richard Middlemore . . . . ib.

13. A Practical Treatise on the Diseases of the Eye. By W. Mackenzie, m.d. To which is prefixed an Anatomical Introduction, explanatory of a Horizontal Section of the Human Eyeball. By Thomas Wharton Jones . . ib.

14. Manuel Pratique des Maladies des Yeux, d'après les Leçons Cliniques de M. le Professeur Velpeau. Par Gustave Jeanselme . . ib.

A Practical Manual of the Diseases of the Eyes, composed from the Clinical Lectures of Professor Velpeau. By Gustavus Jeanselme.

§

4287

PAGE

15. Traité de Pathologie externe et de Médecine opératoire. Par Aug. Vidal (de Cassis) . . . . . . . . 2
A Treatise on Surgical Pathology and on Operative Surgery. By Aug. Vidal (de Cassis).

Art. II.—A Treatise on the Causes and Consequences of Habitual Constipation. By John Burne, m.d. . . . . . . . 40

Art. III.—Clinique Chirurgicale de l'Hôpital d'Instruction de Strasbourg. Par P. Malle . . . . . . . . 49
Clinical Observations in Surgery made in the Hospital of Instruction at Strasbourg. By P. Malle.

Art. IV.—Traité Philosophique de Médecine Pratique. Par A. N. Gendrin, d.m. 55

A Philosophical Treatise on Practical Medicine. By A. N. Gendrin, m.d.

Art. V.—On the Anatomy of the Breast. By Sir Astley P. Cooper, Bart. . 101

Art. VI.—1. The Medical Jurisprudence of Insanity. By J. M. Pagan, m.d. . 129
2. A Treatise on the Medical Jurisprudence of Insanity. By J. Ray, m.d. With an Introductory Essay by D. Spillan, m.d. . . . ib.
3. Du Suicide, de l'Aliénation Mentale, et des Crimes contre les Personnes. Par J. B. Cazauvieilh, d.m. . . . . . . ib.
On Suicide, Insanity, and Crimes against the Person. By J. B. Cazauvieilh.
4. A Letter to the Right Honorable the Lord Chancellor, on the present state of the Law of Lunacy; with Suggestions for its Amendment. By a Barrister of the Inner Temple . . . . . . . ib.
5. De la Folie, considérée dans ses Rapports avec les Questions Medico-judiciaires. Par C. C. H. Marc . . . . . . . ib.
Insanity considered in its Medico-legal relations. By C. C. H. Marc.

Art. VII.—Traité des Etudes Médicales, ou de la Manière d'étudier et d'enseigner la Médecine. Par E. Fred. Dubois (d'Amiens) . . . 175
A Treatise on Medical Study, or on the Method of Studying and of Teaching Medicine. By E. Fred. Dubois (of Amiens).
2. Observations on Medical Education, with a view to Legislative Interference. By Richard Jones, m.r.c.s., &c. . . . . . ib.

Art. VIII.—1. Adversaria Medico-Clinica. Edidit F. G. Lippich, m.d. . 204

Medico-Clinical Notes. By F. G. Lippich, m.d.

Art. IX.—Odontography; or a Treatise on the Comparative Anatomy of the Teeth; their Physiological Relations, Mode of Development, and Microscopic Structure, in the Vertebrate Animals. By Richard Owen, f.r.s. . . 208

Art. X.—Gatherings from Grave-yards; particularly those of London; with a Concise History of the Modes of Interment among different Nations, from the earliest periods. And a Detail of Dangerous and Fatal Results produced by the unwise and revolting custom of Inhuming the Dead in the midst of the Living. By G. A. Walker . . . . . . 212

Art. XI.—Medical Etiquette; or an Essay upon the Laws and Regulations which ought to govern the conduct of Members of the Medical Profession in their relation to each other, &c. By Abraham Banks, m.r.c.s.l. . 215

Art. XII. A Popular Treatise on the Kidney. By George Corfe . . 221

Art. XIII.—On the Preparations of the Indian Hemp, or Gunjah (Cannabis Indica), their Effects on the Animal System in Health, and their Utility in the Treatment of Tetanus and other Convulsive Diseases. By W. B. O'Shaughnessy, m.d. . . . . . . 225

Art. XIV.—De Necessitate Aëris Atmosphericæ ad Evolutionem Pulli in Ovo incubito. Dissertatio Inauguralis Physiologica. Auctore Theod. Schwann 229
On the Necessity of Atmospheric Air for the Evolution of the Chick in the Incubated Egg. By Th. Schwann.

Art. XV.—The Library of Medicine. Arranged and edited by A. Tweedie, m.d. 231

Art XVI.—On the Nature and Structural Characteristics of Cancer, and of those Morbid Growths which may be confounded with it. By J. Müller, m.d. . 235

Art. XVII.—Delle Dottrine sulla Struttura e sulle Funzioni del Cuore e delle Arterie, che imparò per la prima volta in Padova Guglielmo Harvey da

PAGE
Eustachio Rudio, e come essse lo guidarano direttamente a studiare, conoscere, e dimostrare la Circolazione del Sangue. Disquizione di GIOVANNI MARIA ZECCHINELLI . . . . . . 238
On the Doctrines respecting the Structure and Functions of the Heart and Arteries, first learned from Eustachio Rudio by William Harvey, and which were his direct guide to the study, recognition, and demonstration of the Circulation of the Blood. A Dissertation by GIOVANNI MARIA ZECCHINELLI.

### PART SECOND.—Bibliographical Notices.

ART. I.—A Treatise on the Physiological and Moral Management of Infancy. By ANDREW COMBE, M.D. . . . . . 241

ART. II.—First Lines of Education. By EDWARD ASTBURY TURLEY . . 243

ART. III.—Treatise on the Ear. By JOSEPH WILLIAMS, M.D. . . 244

ART. IV.—Mercury, Blue Pill, and Calomel, their use and abuse. By G. G. SIGMOND 245

ART. V.—A Treatise on Amaurosis and Amaurotic Affections. By E. O. HOCKEN 246

ART. VI.—On Diseases of the Bladder and Prostate Gland. By W. COULSON . 247

ART. VII.—A Memoir on Extra-Uterine Gestation. By Dr. W. CAMPBELL . ib.

ART. VIII.—The Principles of Botany. By W. HUGHES WILLSHIRE, M.D. . 248

ART. IX.—A Report on the Progress of Vegetable Physiology during the year 1837. By J. F. MEYEN . . . . . ib.

ART. X.—Pathological Observations on the Diseases of the Uterus. By R. LEE, M.D. 249

ART. XI.—Commentaries on Diseases of the Skin. By A. T. THOMPSON . 250

ART. XII.—Vier Abbildungen des Schädels der Simia Satyrus, von verschiedenen Alter, zur Erklärung der Fable vom Oran Utan. Herausgegeben von Dr. C. F. HEUSINGER . . . . . . 251

Four Representations of the Skull of the Simia Satyrus, at different Periods of Life, with the view of illustrating the Fable of the Orang Utang. By Dr. C. F. HEUSINGER.

### PART THIRD.—Selections from the British and Foreign Journals.

### I. THE FOREIGN JOURNALS.

#### ANATOMY AND PHYSIOLOGY.

M. BAILLARGER's Researches on the Structure of the Cortical Layer of the Convolutions of the Brain . . . . . . 253
M. FAESEBECK on a Peculiar Monstrosity . . . . 254
Dr. GRUBE on the Structure of the Macula Lutea of the Human Eye . . ib.
M. VELPEAU's Notice of a New Monstrosity . . . . 255
Professor Müller on the Lymphatic Hearts of Tortoises . . . 256
Dr. BUROW on the Microscopic Characters of the Menstrual Blood . . 257
M. FLOURENS' Novel Researches concerning the Action of Madder on the Bones ib.
Dr. NASSE on Dr. MARSHALL HALL's Doctrine of the non-participation of Sensation in the (so-called) Reflex Motions . . . . ib.
Professor GLUGE's Microscopical researches on Softening of the Brain . . 259
Dr. TOURTUAL on the Formation of Muscular Fibres, &c. . . . 260

#### PATHOLOGY, PRACTICAL MEDICINE, AND THERAPEUTICS.

M. DUFRESSE on Neuralgia cured by Compression of the Carotid Arteries . 262
M. PELLETAN's Statistical Researches on Pneumonia . . . ib.

#### SURGERY.

Dr. DA LUZ on a Calculus weighing 23½ ounces formed in the Urethra . . 264

Professor REGNOLI on Extirpation of nearly the whole of the Clavicle . 265

M. GUERIN on Subcutaneous Wounds . . . . . ib.

PAGE
M. Berard's Remarkable Case of Hernia of the Fallopian Tube . . 267
Dr. Lyncker on Reduction of Strangulated Hernia by the method of Hesselbach 268
M. Barthelemy on Amputation of the Penis . . . . ib.
Dr. Landouzy on the Facial Hemiplegia of Newly-born Children . . 269
M. Ricord's New Method for the radical Cure of Varia, and especially of Varicocele 270
M. Sedillot's New Plan for Amputating through the Tarsus . . . 271

MIDWIFERY.

M. Dubois's Case of a Dwarf in whom Premature Labour was artificially induced 272
Dr. Dietrich on the Funic Bellows-sound . . . . 274
Dr. Ehrenreich on Compression of the Abdominal Aorta in cases of Flooding . ib.
Dr. Levy on Trismus in New-born Infants . . . . 275

STATISTICS.

Dr. Lohmeyer on Revaccination in the Prussian Army in 1839 . . 276

### II. THE AMERICAN AND COLONIAL JOURNALS.

PATHOLOGY, PRACTICAL MEDICINE, AND THERAPEUTICS.

Dr. Annan's Case of Idiocy from a Blow on the Head . . . 277

Dr. Robertson's Remarks on the Black Vomit of Yellow Fever . . 278

On the Use of Quinine in Yellow Fever . . . . ib.

Dr. Kirkbride's Observations on the Employment of Cimicifuga in Chorea . 280

Dr. Winchester on Smallpox in the Camel .. . . . 281

SURGERY.

Dr. Kearney's Operation for remedying an Anchylosis of the Hip-joint . 282
Dr. Hayward's Case of Vesico-vaginal Fistula, successfully treated by an Operation ib.
Downes on the Treatment of Ulcers in India . . . . 284
Professor Warren on the Removal of the Upper Jaw . . . ib.

### III. THE BRITISH JOURNALS.

Dr. West on Typhus as an Exanthematous Fever . . . . 285
Dr. Smith's Practical Observations on the Diseases of Peru . . ib.
Kerr's Remarks on Collapse in the Treatment of Acute Pneumonic Diseases . ib.
Dr. Paterson's Account of the Rötheln of German Authors . . ib.
Dr. Doherty on the Position of the Placenta . . . . ib.
Dr. Fleming's Practical Observations on Peculiar Affections of the Throat . 286
Halpin on a new Method of Treating Retroversion of the Uterus . . ib.
Appendix to Dr. Graves's Report on the Progress of Cholera . . ib.
Dr. Cane's Observations on Plastic Bronchitis, or Bronchial Polypi . . ib.
Dr. Law on Disease of the Brain, dependent on Disease of the Heart . ib
Dr. Greene's Observations on Empyema . . . . . 287
Smallpox Pustule in the Bladder . . . . . ib.
Dr. O'Beirne's Treatment of Disease of the Hip-joint by Salivation with Mercury ib.
Dr. Mackenzie on the Hydrated Sesquioxide of Iron as an Antidote to Arsenic . ib.
Duffin on a New Mode of Treating Incontinence of Urine during Sleep . ib.
Henderson's Cure for Corns . . . . . ib.
Lucas's Cases of the Cure of Squinting . . . . 288
Mr. Liston on the Cure of Squinting . . . . . ib.
Dr. Bird's Observations on the Employment of Elaterium in Medicine . ib.
Guy on the Frequency of the Pulse at Different Ages in Males and Females . ib.
Dr. Henderson on Acetate of Lead in Bronchitis . . . . ib.
Dr. Julius's Account of Laura Bridgman, an American Girl, with only one Sense ib.

---

### Part Fourth.—Medical Intelligence.

New Bills of Mortality for London . . . . . 289
Medical Association of Ireland . . . . . 292
Provincial Medical and Surgical Association . . . 293
Dr. Foville on the Structure of the Brain . . . 296
Medical Reform . . . . . . 297
Abolition of the practice of Variolous Inoculation . . . 300
Books received for Review . . . . . . ib.

